# Nilotinib as a Prospective Treatment for Alzheimer’s Disease: Effect on Proteins Involved in Neurodegeneration and Neuronal Homeostasis

**DOI:** 10.3390/life14101241

**Published:** 2024-09-28

**Authors:** Ankita Srivastava, Heather A. Renna, Maryann Johnson, Katie Sheehan, Saba Ahmed, Thomas Palaia, Aaron Pinkhasov, Irving H. Gomolin, Thomas Wisniewski, Joshua De Leon, Allison B. Reiss

**Affiliations:** 1 Department Foundations of Medicine, NYU Grossman Long Island School of Medicine, Mineola, NY 11501, USA; ankita.srivastava@nyulangone.org (A.S.); heather.renna@nyulangone.org (H.A.R.); johnson.maryann10@gmail.com (M.J.); ksheehan221@gmail.com (K.S.); saba.ahmed@nyulangone.org (S.A.); thomas.palaia@nyulangone.org (T.P.); 2 Department of Medicine, NYU Grossman Long Island School of Medicine, Mineola, NY 11501, USA; aron.pinkhasov@nyulangone.org (A.P.); irving.gomolin@nyulangone.org (I.H.G.); joshua.deleon@nyulangone.org (J.D.L.); 3 Department of Neurology, Center for Cognitive Neurology, Grossman School of Medicine, New York University, New York, NY 10016, USA; thomas.wisniewski@nyulangone.org

**Keywords:** nilotinib, tyrosine kinase inhibitor, Alzheimer’s disease, amyloid beta, mitochondria

## Abstract

Nilotinib, a tyrosine kinase inhibitor that targets the Abelson tyrosine kinase (c-Abl) signaling pathway, is FDA-approved to treat chronic myeloid leukemia. Nilotinib has properties indicative of a possible utility in neuroprotection that have prompted exploration of repurposing the drug for the treatment of Alzheimer’s disease (AD) and Parkinson’s disease (PD). AD is a progressive age-related neurodegenerative disorder characterized by the deposition of extracellular amyloid-β plaques and intracellular neurofibrillary tangles. It is incurable and affects approximately 50 million patients worldwide. Nilotinib reduces c-Abl phosphorylation, amyloid-β levels, and dopaminergic neuron degeneration in preclinical AD models. This study explores the effects of nilotinib on amyloid processing and mitochondrial functioning in the SH-SY5Y human neuroblastoma cell line. SH-SY5Y cells were exposed to nilotinib (1, 5, and 10 µM). Real-time PCR and immunoblot analysis were performed to quantify the expression of genes pertaining to amyloid-β processing and neuronal health. Nilotinib did not significantly change APP, BACE1, or ADAM10 mRNA levels. However, BACE1 protein was significantly increased at 1 µM, and ADAM10 was increased at 10 µM nilotinib without affecting APP protein expression. Further, nilotinib treatment did not affect the expression of genes associated with neuronal health and mitochondrial functioning. Taken together, our findings do not support the efficacy of nilotinib treatment for neuroprotection.

## 1. Introduction

The treatment of neurodegenerative diseases such as Alzheimer’s disease (AD), Parkinson’s disease (PD), and others has proven extremely challenging, with no cure and limited success in slowing progression [1,2]. AD is an irreversible neurodegenerative disorder and the most common form of dementia affecting people over the age of 65 years [3,4]. For decades, the deposition of extracellular amyloid-β (Aβ) plaque and intracellular neurofibrillary tangles of tau protein have been the focus of AD research [5]. However, this narrow focus has led to the failure of numerous clinical trials applying immunotherapy against these misfolded proteins [6,7]. There is still controversy in the efficacy of new Aβ-targeting drugs that successfully reduce the accumulation of Aβ and progression of AD [8]. Despite the increasing incidence of AD as the global population ages, there is no curative therapy. Thus, studies have been focusing on new molecular targets that regulate different cellular pathways in pertinent regions of the brain.

In an effort to develop novel approaches to AD treatment, attention has recently been focused on several tyrosine kinases, including the members of the Abelson (Abl) tyrosine kinase and discoidin domain receptor (DDR) families that are upregulated in AD patients [9,10]. The activation of both Abl and DDR signaling causes disturbances in synaptic plasticity, increases oxidative stress, alters homeostatic molecular mechanisms, and eventually causes neuronal death [11]. The c-Abl protein is a non-receptor tyrosine kinase activated in response to oxidative and cellular stress [12]. Its activation is associated with the accumulation of the alpha-synuclein protein and the deactivation of the Parkin protein, and it is therefore of interest as a target for the treatment of Parkinson’s disease and, more recently, AD. The discoidin domain receptors, DDR1 and DDR2, are members of the receptor tyrosine kinase family that bind to extracellular collagens and modulate cell growth and survival [13].

Nilotinib is a second-generation oral c-Abl tyrosine kinase inhibitor that acts by occupying the ATP binding domain in ABL, thus preventing substrate phosphorylation. It is used clinically to treat Philadelphia chromosome positive chronic myelogenous leukemia [14,15]. Nilotinib also inhibits DDR1 and DDR2, receptor tyrosine kinases involved in cellular differentiation and proliferation and in extracellular matrix remodeling [16]. The inhibition of DDR1 by nilotinib may reduce neuroinflammation [17]. Nilotinib-mediated c-Abl inhibition is being evaluated as a therapeutic strategy to rescue cells from intraneuronal amyloid and tau toxicity [11,18]. c-Abl plays an important role in the pathogenesis of neuroinflammation and neurodegeneration associated with AD [19]. 

The basis for studying nilotinib as a neuroprotective began with animal models of PD in which the drug prevented neuronal loss [20]. Wu et al. used a model of neuroinflammation induced by lipopolysaccharide (LPS) injection into the ventral midbrain to show that nilotinib treatment could lessen microglial activation and prevent injury to dopaminergic neurons by reducing the microglial release of inflammatory cytokines [21]. This led to human PD studies with no clinical benefit thus far, but good safety and tolerability [22,23,24]. 

In animal models, nilotinib can cross the blood–brain barrier and incite the autophagic clearance of amyloid, tau, and other neurotoxic proteins [25,26,27,28]. In addition to the promotion of the clearance of misfolded proteins, the high levels of c-Abl activation in AD mice and human AD and PD brain tissue prompted the study of nilotinib in AD [29,30,31]. 

In light of the ongoing clinical trials exploring nilotinib therapy for neurodegenerative disorders, the goal of the present study is to determine the potential role of nilotinib in neuronal health and mitochondrial functioning using a human neuronal cell culture model. 

## 2. Materials and Methods

### 2.1. Cell Culture and Nilotinib Treatment

Human neuroblastoma SHSY-5Y cells (American Type Culture Collection, Manassas, VA, USA) were cultured in DMEM-F12 culture media. Culture medium was supplemented with 10% fetal bovine serum (FBS), 2 mM L-glutamine, and 50 μg per mL of penicillin-streptomycin at 37 °C in a 5% CO_2_ atmosphere. Cells were seeded at a density of 500,000 cells per mL. Cell culture media and supplementary reagents were obtained from Invitrogen (Grand Island, NY, USA). Cells were treated with 1 µM, 5 µM, and 10 µM of nilotinib (from APExBIO, Houston, TX, USA). After 24 h of nilotinib treatment, cells were used and proceeded for further experiments.

### 2.2. Real-Time PCR

Total RNA was isolated after 24 h of nilotinib treatment using Trizol reagent (Invitrogen, Grand Island, NY, USA) and dissolved in DEPC water (Invitrogen, Grand Island, NY, USA). cDNA was synthesized from 1 µg of total RNA. cDNA was then subsequently used for quantitative real-time PCR analysis on Light Cycler 480 (Roche Diagnostics, Indianapolis, IN, USA) using FastStart SYBR Green Reagents Kit (Roche Diagnostics, Indianapolis, IN, USA) according to the manufacturer’s instructions; glyceraldehyde-3-phosphate dehydrogenase (GAPDH) was used as a housekeeping gene. Analysis of gene expression was obtained by using the (2^−ΔΔCt^) method, normalized to housekeeping gene GAPDH. The list of primers used is given in Table 1.

### 2.3. Western Blotting

Protein samples were collected from whole cell lysates using radioimmunoprecipitation assay (RIPA) lysis buffer (98% PBS, 1% Igepal, 0.5% sodium deoxycholate, 0.1% sodium dodecyl sulfate), supplemented with 10 μL per mL of protease inhibitor cocktail (Sigma, Rockville, MD, USA) after 48 h of nilotinib treatment. Concentration of protein was measured using the BCA Protein Assay Kit (Pierce Biotechnology Inc., Rockford, IL, USA). In total, 8.3 µg of each protein sample was loaded and separated by 8–12% SDS-polyacrylamide gel electrophoresis (SDS-PAGE). Separated proteins were transferred to a polyvinylidene difluoride membrane. The membrane was then subsequently blocked with 5% milk in TBST for 1 h at room temperature and then incubated in primary antibodies at 4 °C overnight. After overnight incubation, the membrane was then incubated with secondary antibodies diluted in 5% milk in TBST. Bound antibodies were visualized with horseradish peroxidase-conjugated secondary antibodies. The immunoreactive protein was detected using ECL Western blotting detection reagents (Thermo Scientific™ SuperSignal™ West Pico PLUS Chemiluminescent Substrate, Waltham, MA, USA) and the Bio-Rad ChemiDoc Touch Imaging System. Loading in each lane was validated using β-actin as an internal loading control. Quantification of protein blots was performed using Image J software version 1.52a.

### 2.4. Transmission Electron Microscopy

Cell culture samples were fixed in 2.5% glutaraldehyde buffered in 0.1 M sodium cacodylate buffer, pH 7.5, washed in 0.1 M sodium cacodylate buffer and post-fixed, and stained with 1% osmium tetroxide buffered in sodium cacodylate. Cells were then re-moved from the plastic culture dish, centrifuged, en-bloc stained with a saturated solution of uranyl acetate in 40% ethanol, dehydrated in a graded series of ethanol, infiltrated in propylene oxide with Epon epoxy resin (Embed812, Electron Microscopy Sciences, Hatfield, PA, USA), and embedded. The blocks were sectioned with a Reichert Ultracut microtome at 70 nm. Sections were picked up on 300 mesh copper grids, dried, and then post-stained with a 1% aqueous uranyl acetate followed by 0.5% aqueous lead citrate. Stained grids were examined on a Zeiss EM 900 transmission electron microscope retrofitted with an SIA L3C digital camera (SIA, Duluth, GA, USA).

### 2.5. MitoTracker Staining

SH-SY5Y cells were stained with MitoTracker dye that stains active mitochondria in live cells. Cells were seeded in a 6-well plate and treated with 1, 5, and 10 µM of nilotinib. After 48 h of nilotinib treatment, cells were washed with Hanks’ Balanced Salt Solution (HBSS) and stained with 250 µM of MitoTracker (Thermo Fisher Scientific, M7512, Waltham, MA, USA) for 20 min. After staining, cells were washed three times with HBSS. Images were captured at 10× using fluorescence microscope. 

### 2.6. Statistical Analysis

Data were represented as mean ± standard deviation (SD). Statistical significance was analyzed by one-way ANOVA followed by Bonferroni’s multiple comparison test. Data were analyzed on GraphPad Prism version 9.2.0 (332) (GraphPad Software, La Jolla, CA, USA). Fold change was calculated between control and treated groups. *p* values < 0.05 were considered significant.

## 3. Results

### 3.1. Effect of Nilotinib on Genes Regulating Amyloid-β Formation in SH-SY5Y Cells

**Figure 1:**
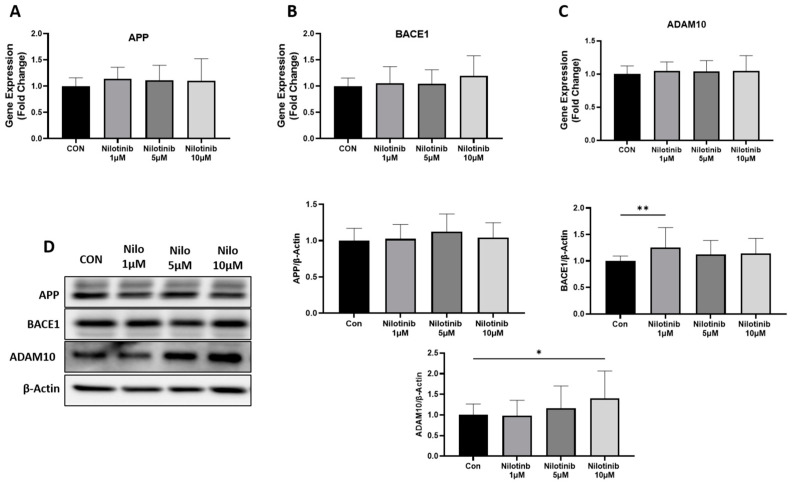
Effect of nilotinib treatment on APP, BACE1, and ADAM10 expression in SH-SY5Y cells. (**A**–**C**) Real-time PCR analysis of APP, BACE1, and ADAM10 in SH-SY5Y cells after nilotinib treatment. GAPDH was used as an internal control. (**D**) Western blot analysis of APP, BACE1, and ADAM10 in SH-SY5Y cells after nilotinib treatment. Densitometry of representative blot was normalized with β-actin. Data were represented in fold difference. N = 6–9, ** *p* < 0.01, * *p* < 0.05 based on a one-way ANOVA followed by Bonferroni multi-comparison tests.

To investigate the role of nilotinib in regulating Aβ formation, we measured the expression of genes involved in Aβ formation such as APP, BACE1, and ADAM10 in SH-SY5Y cells at both the mRNA and protein level after nilotinib exposure. Compared to the control, the treatment of nilotinib at different concentrations (1 µM, 5 µM, and 10 µM) did not change the mRNA levels of APP, BACE1, and ADAM10 in SH-SY5Y cells at any of the nilotinib concentrations (Figure 1A–C). Further, we checked the expression of APP, BACE1, and ADAM10 at the protein level in SH-SY5Y cells. The exposure of nilotinib did not change the protein levels of APP at any concentration, but the protein levels of BACE1 and ADAM10 were significantly increased at 1 µM and 10 µM of nilotinib concentration, respectively (Figure 1D). 

### 3.2. Effect of Nilotinib on Genes Associated with Neuronal Health in SH-SY5Y Cells

**Figure 2:**
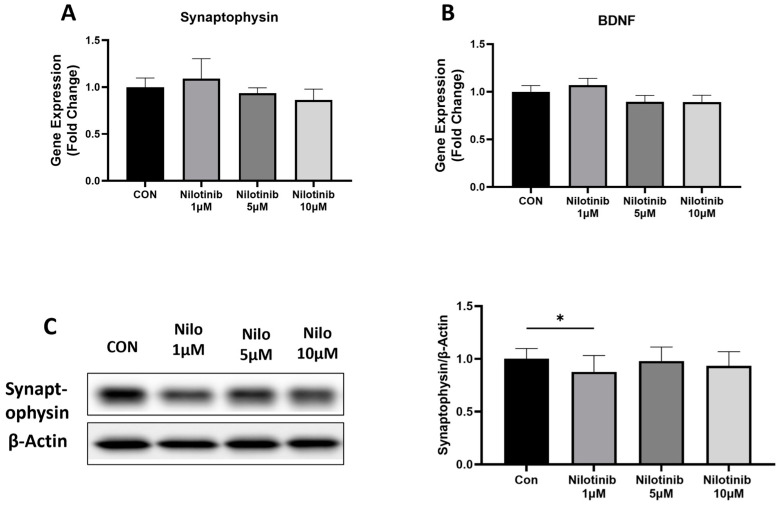
Effect of nilotinib treatment on expression of genes involved in neuronal health. (**A**,**B**) Real-time PCR analysis of synaptophysin and BDNF in SH-SY5Y cells after nilotinib treatment. GAPDH was used as an internal control. (**C**) Western blot analysis of synaptophysin in SH-SY5Y cells after nilotinib treatment. Densitometry of representative blot was normalized with β-actin. Data were represented in fold difference. N = 6–9, * *p* < 0.05, based on a one-way ANOVA followed by Bonferroni multi-comparison tests.

To determine the neuronal health of SH-SY5Y cells following nilotinib treatment, we quantified the expression of synaptophysin and BDNF at the mRNA level and synaptophysin at the protein level. Real-time PCR analysis showed no change in the mRNA levels of synaptophysin and BDNF at any concentration of nilotinib compared to the control in SH-SY5Y cells (Figure 2A,B). Further, we checked the expression of synaptophysin at the protein level and found a significant decrease in synaptophysin protein level at 1 µM concentration of nilotinib in SH-SY5Y cells (Figure 2C).

### 3.3. Effect of Nilotinib on Mitochondrial Functioning in SH-SY5Y Cells

**Figure 3:**
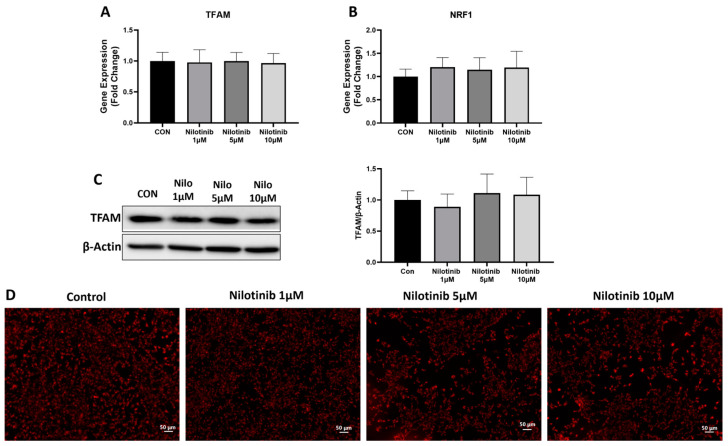
Effect of nilotinib treatment on expression of genes associated with mitochondrial functioning. (**A**,**B**) Real-time PCR analysis of TFAM and NRF1 in SH-SY5Y cells after nilotinib treatment. GAPDH was used as an internal control. (**C**) Western blot analysis of TFAM in SH-SY5Y cells after nilotinib treatment. Densitometry of representative blot was normalized with β-actin. (**D**) Microscopic images of live SH-SY5Y cells stained with 250 µM of MitoTracker dye after nilotinib treatment. Scale bar: 50 µm. Data were represented in fold difference. N = 6–9, significance was calculated using one-way ANOVA followed by Bonferroni multi-comparison tests.

To characterize the markers of mitochondrial functioning, we measured the expression of mitochondrial transcription factor A (TFAM) and nuclear respiratory factor 1 (NRF1) genes. First, we checked the expression of TFAM and NRF1 at the mRNA level and found that TFAM and NRF1 mRNA levels did not change with nilotinib treatment as compared to control conditions in SH-SY5Y cells (Figure 3A,B). We further measured the protein levels of TFAM after nilotinib treatment and found no difference in TFAM protein levels as compared to the control after nilotinib treatment in SH-SY5Y cells (Figure 3C). We also performed MitoTracker staining to stain active mitochondria in SH-SY5Y cells. Staining images showed no difference in active mitochondria in SH-SY5Y cells after nilotinib treatment as compared to the control condition (Figure 3D). 

### 3.4. Effect of Nilotinib on Mitochondrial Morphology in SH-SY5Y Cells

**Figure 4:**
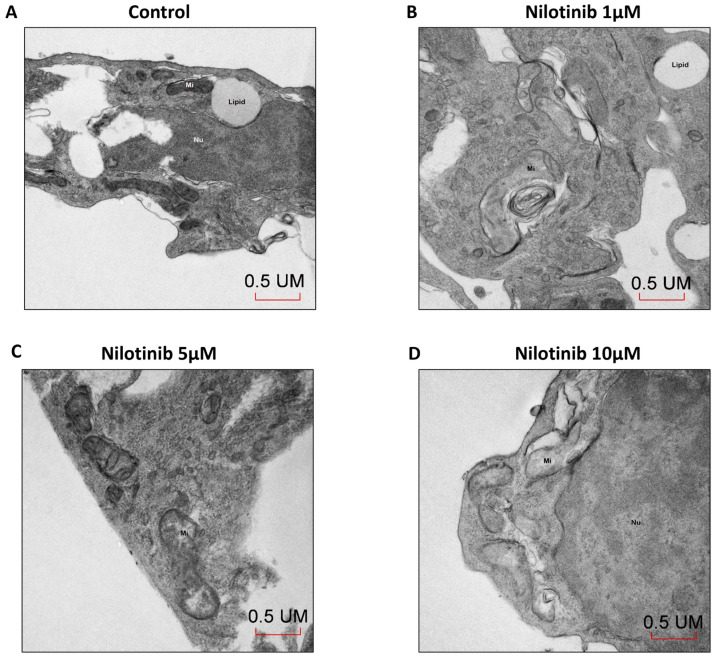
Effect of nilotinib treatment on mitochondrial morphology. (**A**–**D**) Representative TEM images of SH-SY5Y cells treated with 0, 5, and 10 µM concentrations of nilotinib for 48 h. All images were taken at 30,000× magnification. Scale bar: 0.5microns (UM).

To determine mitochondrial morphology, transmission electron microscopy (TEM) was completed in SH-SY5Y cells after 48 h of nilotinib treatment (Figure 4). TEM images showed that nilotinib treatment in SH-SY5Y cells resulted in dysfunctional mitochondria as compared to control cells. With increasing doses of nilotinib, mitochondrial profiles became progressively enlarged with a more washed-out matrix and fewer disorganized cristae (Figure 4).

## 4. Discussion

In this study, the possible neuroprotective properties of nilotinib were evaluated in SH-SY5Y human neuroblastoma cells. Two major hallmarks, amyloid processing and mitochondrial functioning, play important roles in the pathogenesis of AD and other neurodegenerative disorders such as synucleinopathies [32], and can be used as surrogate markers for the assessment of the neuroprotective effects of treatment. Our data show that exposure of SH-SY5Y cells to nilotinib did not change APP levels, but did impact BACE1 and ADAM10. BACE1 protein was upregulated by the 1 µM dose, which would be amyloidogenic, while ADAM10 was increased at the 10 µM dose, which would be non-amyloidogenic. In addition, we did not find any difference in BDNF expression, but synaptophysin protein levels went down at the 1 µM dose of nilotinib. We also checked mitochondrial functioning and did not find any difference after nilotinib exposure. To our knowledge, this is the first study attempting to understand the mechanisms of nilotinib effects on neuronal health in human SH-SY5Y cells.

Several pharmacological approaches against tyrosine kinase in cancer research provide a promising strategy involving drug repurposing against neurodegenerative diseases as well. Thus, cancer treatments provide clues to a possible method for the treatment of AD and nilotinib; a c-Abl inhibitor seems to be an interesting approach in the treatment of neurodegenerative diseases such as PD and perhaps AD [11,12,21,33,34]. Nilotinib targets c-Abl and DDRs and is used for the treatment of chronic myeloid leukemia positive for the Philadelphia chromosome. Studies showed that c-Abl and DDRs are upregulated in neurodegenerative diseases such as PD and AD. The inhibition of these in in vitro and in vivo experimental models of AD provides a rationale for using nilotinib in AD treatment [35,36,37]. Nilotinib enhanced parkin-Beclin-1 interaction, resulting in Aβ clearance and improved cognitive performance in an animal model of AD [25]. La Barbera et al., 2021, reported that nilotinib reduced c-Abl phosphorylation and Aβ levels as well and prevented the degeneration of dopaminergic neurons in the Tg2576 murine model of AD [35]. 

A recent study showed that the inhibition of CNS c-Abl upon nilotinib treatment reduced Aβ load and tau phosphorylation in AD experimental models and suggested nilotinib as a suitable preclinical candidate for AD therapies [38]. Currently, nilotinib is under clinical trials to determine its therapeutic potential in patients suffering from AD. The promising results from a Phase II trial in reducing Aβ and bringing about cognition improvement have encouraged the scientific community to move on to Phase III trials with high expectations. According to the Phase II study conducted by Turner et al., 2020, nilotinib is safe in AD patients with mild-to-moderate dementia. The researchers found that nilotinib reduced the amyloid burden in the frontal lobe of AD patients, cerebrospinal fluid Aβ40 was reduced at 6 months, and Aβ42 was reduced at 12 months in the nilotinib-treated group as compared to the placebo group [11]. 

It is well established that both differentiated and undifferentiated SH-SY5Y cells can be used to determine neuronal health. In our previously published study, we showed the importance of P110 in undifferentiated SH-SY5Y cells and its protective role in regulating Aβ formation and mitochondrial functioning [39]. In the current study, we used undifferentiated SH-SY5Y cells to evaluate the role of nilotinib in neuronal health. 

Experimental studies with therapies aiming to reduce Aβ accumulation in the brain of AD preclinical models often do not translate to humans [6]. The new anti-amyloid drugs approved by the Food and Drug Administration have shown some modest efficacy, but are controversial [40,41,42]. Nilotinib has been shown in one clinical trial to reduce amyloid burden in mild-to-moderate AD patients [11]. On this basis, the anti-amyloid effects of nilotinib would be magnitudes less than the drugs now on the market and unlikely to be nilotinib’s primary mode of action if it were effective. The biochemical alteration in Aβ accumulation is one of the core hallmarks of AD pathogenesis, but more and more data are accumulating to indicate that Aβ is not the causal mechanism of AD [43,44,45,46]. 

The production of Aβ from APP is initiated by BACE1, a major β-secretase enzyme. Our recently published study showed the reduction in APP and BACE1 protein levels in SH-SY5Y cells upon exposure to P110 [39]. Using microRNA to target the amyloidogenic pathway, the regulatory role of BACE1 and APP has been demonstrated in experimental models of AD utilizing mice that have been manipulated to accumulate amyloid in the brain as their primary pathology [47]. However, human AD is much more complex and, while a human study showed an association between BACE1 and a BACE1-targeting microRNA in AD, the value of the BACE1 measure was as a biomarker and not therapeutic [48]. In contrast to BACE, ADAM10, an α-secretase, is responsible for the cleavage of APP through a non-amyloidogenic pathway; thus, ADAM10 serves as a suitable target for reducing the accumulation of Aβ plaques [49]. However, the full mechanism of increased BACE1 and ADAM10 expression without any change in APP levels needs to be elucidated and thus requires more experiments to create a complete picture. 

In addition to the anti-amyloidogenic property, neuroprotective properties are likely even more important in determining the therapeutic effect of an AD drug. Our previous research showed the anti-amyloidogenic as well as neuroprotective effect of P110 in SH-SY5Y cells [39]. BDNF shows a neuroprotective effect and slows down the progression of neurodegeneration, thus serving as a promising target for AD intervention [50,51]. To our knowledge, this is the first study to determine the role of nilotinib in the expression of neuroprotective genes that are relevant to cognitive function. The exposure of SH-SY5Y cells to nilotinib did not change BDNF expression, but reduced synaptophysin expression. Previous studies have reported the loss of the presynaptic marker synaptophysin in AD models [52,53]. Thus, our results indicate that nilotinib did not have any effect on genes associated with neuronal health in SH-SY5Y cells, but further comprehensive evaluation is necessary to form a complete picture of nilotinib and its effect on neuronal health in AD models.

Dysfunction in mitochondria is the key mediator of AD pathogenesis. Most of the clinical trial studies still focus on the classical targets of AD (Aβ, tau, ApoE), but there are novel research boosts to study mitochondria and bioenergetics as an alternative strategy for the treatment of AD. Mitochondrial dysfunction in AD is related to increased Aβ production, the hyperphosphorylation of tau, neuro-inflammation, the elevated production of reactive oxygen species (ROS), neuronal apoptosis, reduced mitochondrial biogenesis, and impaired brain metabolism [54,55]. One meta-analysis study suggested that neuroinflammation and oxidative stress associated with mitochondrial dysfunction may occur in the early stage of AD progression [46]. A study conducted by Yao et al. showed mitochondrial dysfunction in transgenic AD mice at 3 months of age, while mitochondrial Aβ levels were increased at 9 months [45]. Thus, targeting mitochondrial bioenergetics serves as a promising approach of AD treatment. 

Under normal conditions, mitochondrial fission and fusion are balanced, whereas in AD this balance is compromised due to alterations in the expression of mitochondrial fission and fusion proteins. The consequence is impaired mitochondrial morphology and biogenesis [56,57]. Calkins et al. showed the deposition of Aβ plaque in mitochondria impaired mitochondrial movement at neuronal synapses, leading to the degeneration of synapses in AD neurons [58]. A study in transgenic mice and AD brains showed that the accumulation of APP/Aβ in the mitochondrial membrane affects mitochondrial function via the attenuation of the activity of electron transport chain enzymes and reduction in oxygen consumption rate [59]. Our study reveals that the exposure of SH-SY5Y cells to nilotinib did not change mitochondrial biogenesis, and this is consistent with our gene expression data and microscopy images, thus suggesting no improvement of mitochondrial functioning after nilotinib treatment. In fact, TEM demonstrated detrimental structural changes in the mitochondria with nilotinib. 

This study has some limitations as our experiments were all performed in cell cultures and further studies are required to analyze the effect of nilotinib on neuronal health in primary human neurons and in vitro and in vivo AD models. Additional research is needed to elucidate mitochondrial activity and ultrastructural changes upon nilotinib treatment, particularly if efficacy is detected in human clinical trials.

## 5. Conclusions

Our findings in a human neuronal cell model did not show any impact of nilotinib in key aspects of neuroprotection. In this study, we have shown that the drug does not modify the expression of genes involved in amyloid processing and mitochondrial functioning, two major hallmarks of neuronal health. Further investigation to determine possible mechanisms of neuroprotection with nilotinib using neurons derived from AD subjects is warranted as we await the results of clinical trials.

## Figures and Tables

**Table 1:** Human primer sequences with Tm for real-time PCR.

Primer (Tm)	Forward Sequence	Reverse Sequence
GAPDH (62 °C)	ACCATCATCCCTGCCTCTAC	CCTGTTGCTGTAGCCAAAT
APP (62 °C)	TTTGGCACTGCTCCTGCT	CCACAGAACATGGCAATC
BACE-1 (62 °C)	GCAGGGCTACTACGTGGAGA	CAGCACCCACTGCAAAGTTA
TFAM (63 °C)	AAGATTCCAAGAAGCTAAGGGTGA	CAGAGTCAGACAGATTTTTTCCAGTTT
SYNAPTOPHYSIN (65 °C)	CTGCAATGGGTCTTCGCCA	ACTCTCGGTCTTGTTGGC
ADAM10 (62 °C)	TCGAACCATCACCCTGCAACCT	GCCCACCAATGAGCCACAATCC
NRF-1 (63 °C)	GGCACTGTCTCACTTATCCAGGTT	CAGCCACGGCAGAATAATTCA
BDNF (64 °C)	AGCTATCCAGAGCATCTTCCA	ACCTGGTGGAACTTTATGAAACC

Abbreviations: ADAM10—a disintegrin and metalloproteinase domain-containing protein 10; APP—amyloid precursor protein; BACE-1—β-secretase-1; BDNF—brain-derived neurotrophic factor; GAPDH—glyceraldehyde-3-phosphate dehydrogenase; NRF-1—nuclear respiratory factor 1; TFAM—mitochondrial transcription factor A.

## Data Availability

The full dataset is available from the corresponding author upon motivated request.
